# Denudation of human amniotic membrane by a novel process and its characterisations for biomedical applications

**DOI:** 10.1007/s40204-016-0053-7

**Published:** 2016-08-01

**Authors:** R. Sripriya, R. Kumar

**Affiliations:** 1 Sree Balaji Medical College and Hospital, Bharath University, Chennai, India; 2 Department of Life Sciences (R&D), Datt Mediproducts Ltd., Gurgaon, India

**Keywords:** FT-IR, DSC, SEM, Histology, Proliferation, Limbal cells

## Abstract

This study was aimed to investigate the suitability of a modified method to get decellularised human amniotic membrane (DHAM). The obtained membrane was subjected to physico-chemical and biological evaluations to validate its potential for biomedical applications. The human amniotic membrane was processed with detergent and alkali followed by enzymatic treatments. Hematoxylin and eosin (H&E) and Masson’s trichrome staining of membrane were in accordance with conjectures: the decellularised membrane stained for extracellular matrix is rich in collagen. Scanning electron micrograph also showed the denudation in the processed membrane with the cellular impressions on the basement membrane. Physical characteristics namely the differential scanning calorimetric, tensile, shrinkage behaviour and the Fourier transform infrared spectra of decellularised membrane showed its stability and intact structure similar to the unprocessed membrane. In the visible range of light, the membrane was found to be transparent from 90 to 98 %. Proliferation rate of fibroblasts, keratinocytes, myoblasts and hepatocytes were significantly upregulated compared to the control. The cell morphologies were normal and differentiation of myoblasts into myotubes were more pronounced in decellularised membrane. Proliferation of corneal limbal cells on decellularised membrane showed 92–100 % confluency on day 21 and the migrated cells displayed a spindle shape and changing later to a more cuboidal appearance.

## Introduction

The innermost layer of placenta is the amniotic membrane (AM), the thickness of which ranges from 0.02 to 0.05 mm. It consists of an epithelial layer, basement membrane and avascular stroma. Human AM (HAM) has been used as a biomaterial in the field of dermatology in plastic surgery, skin transplantations and as a biological dressing for skin burns, wounds, chronic leg ulcers, ophthalmic healing, etc. (Gholipourmalekabadi et al. 2015; Davis 1910; Azuara-Blanco et al. 1999; Dua et al. 2004). Other studies have demonstrated the benefits of HAM as a substrate for ex vivo expansion of diverse cell lines such as limbal epithelial, corneal and conjunctival epithelial cells (Grueterich et al. 2002; Pellegrini et al. 1997).

The preservation strategies play a major role in retaining the biomaterial properties of HAM. Cryopreservation and lyophilization are the common techniques of preservation, but are not feasible every time due to their limitations. Moreover, following these techniques HAM will be preserved with epithelium which can be more immunogenic. The same feature can also limit its application for cell culture and tissue regeneration application because migration and differentiation of cultivated cells on HAM can be impeded (Shortt et al. 2009). To overcome this limitation, HAM can be denudated and the same can be processed either chemically (Davis 1910), or enzymatically (Gipson and Grill 1982), or in combination of both (de Melo et al. 2007). Methods of denudation involve removing the cells and cellular debris from the amniotic membrane while leaving the extracellular structural proteins intact. This decellularised, i.e., denudated membrane is considered as “scaffold”.

Effective decellularisation methodology can retain the recellularisation potential of the HAM by retaining its biological property with decreased antigenicity. Previously, numerous decellularisation methodologies have been reported yielding mixed results (Simon et al. 2003; Rieder et al. 2004) following vigorous methodologies to alter the biomechanical and physico-chemical properties of the matrix resulting in a compromised material further limiting its potential applicability in the field of tissue regeneration.

Methods of decellularisation in heart valves report the use of trypsin (Bader et al. 2000) or a non-ionic detergent like triton X-100 followed by ribonuclease digestion (Bader et al. 1998) which leads to incomplete removal of cells. Bader et al. (2000) described that a longer incubation with trypsin might be more effective in cell removal but causes a disruption of the matrix bundle of HAM. Reported methods on human amniotic membrane decellularisation include the use of hypotonic tris buffer, protease inhibitors and nuclease treatment along with sodium dodecyl sulfate (SDS) (Wilshaw et al. 2008) and in some cases, treatment with Dispase II has also been reported (Lim et al. 2009). Though SDS treatment, commonly used for decellularisation was found to have destabilizing effect on collagen triple helix domain it was also responsible to swell the elastin network of the HAM (Samouillan et al. 1999). In the same way, treating HAM with Dispase II leads to the major changes in the basement membrane of HAM in terms of its constituents and ultrastructure which makes the membrane unsuitable for the growth of explant culture (Lim et al. 2009). Treatment of HAM with EDTA and/or proteolytic enzymes; in many cases require additional mechanical and can result in membrane damage (Saghizadeh et al. 2013). To overcome these limitations and to utilise the benefits of this membrane in diverse applications, the appropriate processing and preservation of the membrane is essential.

We have proposed a novel method to decellularise the HAM without affecting the basic structure of extracellular matrix. This decellularised membrane was then cross-linked using UV radiation to improve its stability. The processed decellularised HAM (DHAM) was subjected to various physico-chemical and biological characterizations and the results were compared with the unprocessed HAM and discussed.

## Materials and methods

### HAM acquiring and transportation

HAM were collected form patients undergoing cesarean delivery after screening for treats like HIV 1&2, hepatitis B virus, hepatitis C virus, syphilis and malaria. Membranes were processed under sterile conditions, as previously described by Tseng et al. (1998). The research parameters were performed as per the ethical guidelines, approved by Institutional Ethics Committee (IECs) of Sree Balaji Medical College and Hospital, Chrompet, Chennai, India.

### Denudation of amniotic membrane

The HAM was washed with DMEM (Dulbecco’s modified eagle’s medium) containing antibiotics under the laminar air flow to make it free from blood clots. Any leftover chorion attached to the HAM and blood clots were gently peeled off from the epithelial cell layer using round-ended forceps. The membrane was then trimmed into squares of size 5 × 5 cm^2^ and were spread on top of a nitrocellulose paper with epithelial side upwards, and stored in DMEM containing vials at −80 °C for further studies.

Decellularisation of amniotic membrane was performed using earlier method reported by us with slight modification (Sripriya et al. 2003). To being with, the membrane was treated with (0.2, 0.3, 0.4 and 0.5 % w/v) non-ionic surfactant and then suspended in (0.2, 0.3, 0.4 and 0.5 % w/w) sodium peroxide solution for 3 h followed by repeated washing with distilled water. The membrane was then immersed in 67 mM phosphate buffer (pH 8.5) and treated with trypsin (0.2, 0.3, 0.4 and 0.5 % w/w) for 6, 8, 10 and 12 h. After the enzymatic treatment, tissues were washed repeatedly with water to remove the enzyme. The obtained DHAM was mounted on a glass plate and irradiated with near UV (NUV) light at 365 nm for 3 h and then stored in isopropyl alcohol until use. The processed DHAM was subjected to biological and physico-chemical characterizations.

### Estimation of collagen content

Collagen content of the HAM has been estimated by quantifying the hydroxyproline, using the method of Neuman and Logan (1950).

### Differential scanning calorimetric (DSC) studies

DSC studies of the HAM membrane samples were performed using a calorimeter (universal V4.4A TA instruments) at 24 °C and 65 % R.H. Samples of membranes namely unprocessed and DHAM were shielded in aluminium containers and subjected to heating at the rate of 10 °C per minute in N_2_ atmosphere. The thermograms were recorded in the temperature range between 0 and 300 °C.

### Fourier transform infrared spectroscopy (FTIR) studies

FTIR spectral studies of the amniotic membrane samples were performed by using Nicolet 2DDKB FTIR spectrometer and OMNIC software (Version 6.0). The spectra were recorded in the range of 400–4000 cm^−1^ at 25 °C with 30–100 % transmittance (T).

### Histological analysis

The unprocessed HAM and DHAM samples were fixed in 10 % (v/v) neutral buffered formalin, dehydrated, and then embedded in paraffin wax. Tissue sections (1–3 µm) were stained by hematoxylin and eosin (H&E) and Masson’s trichrome and analysed under a light microscope.

### Scanning electron microscopy

Prior to scanning electron microscopic observations, the HAM specimens were dehydrated in a graded ethanol series of 25, 50, 75, 95 and 100 % each for 10 min. Dehydration in 100 % ethanol was done thrice. The samples were then dried and mounted on SEM stubs using carbon adhesive tabs. They were then sputter coated with a 10 nm thick layer of gold and examined with a scanning electron microscope (JSM-5600; JEOL, Tokyo, Japan).

### Studies of shrinkage temperature

The shrinkage temperatures of the HAM (unprocessed HAM and DHAM) were measured using micro shrinkage meter fitted with a field microscope. The HAM membrane (1 cm^2^, in distilled water) was placed on the wet surface (distilled water) of micro slide and the temperature of the slide was raised slowly (1 °C/min). The shrinkage of the membrane was observed through the microscope. Shrinkage temperature of the unprocessed HAM and DHAM were measured using micro-shrinkage apparatus.

### Studies of tensile strength

Tensile strength was tested by instron series II automated materials testing system. The samples were cut into 16 mm sized dumbbells with 5.15 mm inner width and then immersed in distilled water for 30 min. Tensile force was applied at an extension rate of 10 mm/min. The ends of the sample were held by pneumatic grips (40 psi grip pressure).

### Transparency measurements

The transparency of the denuded amniotic membrane was investigated by recording percentage transmission of the electromagnetic waves ranging from 250 to 700 nm using a UV–visible spectrophotometer. The DHAM was placed over quartz cuvettes and the transmittance was recorded with respect to the air (i.e., control spectrum without sample).

### Biological characterization

The biological characterizations of the HAM membrane were performed by cell proliferation and their morphological examinations using rat cardiac fibroblasts (RCF), NIH3T3 fibroblasts, A431 keratinocytes, L6 myocytes and HepG2 hepatocytes. RCF isolation from cardiomyocytes and subsequent preparation was done as described previously (Chrishan et al. 2009). All the cells were grown and maintained in DMEM containing high glucose with 10 % fetal calf serum (FCS) supplemented with penicillin (120 units/ml), streptomycin (75 mg/ml), gentamycin (160 mg/ml) and amphotericin B (3 mg/ml) at 37 °C in an incubator humidified with 5 % CO_2_. Further validation of DHAM towards human ophthalmic application was performed using human corneal limbal explant culture. The cell viability was monitored by trypan blue and MTT assay (Mossmann 1983).

### Collection of corneal limbal tissue and explant culture

Corneal limbal tissue of 2 mm^3^ (*n* = 6) was collected from the cadaveric donor eye from Eye Bank of medical research foundation, Chennai and processed for further experiments. The excised tissues were washed thrice with PBS containing double strength antibiotics and the epithelial tissues were cut into multiple bits using sterile blade and the bits were transferred to tissue culture plates followed by incubation at 37 °C and 5 % CO2 for 5 min. The culture medium, DMEM-F12 [1:1] containing 50 ng/ml of streptomycin, 1.25 ng/ml of amphotericin B, 2 ng/ml of mouse epidermal growth factor (EGF), 5 ng/ml of insulin, 5 ng/ml of transferrin, 5 ng/ml of selenium, 5 mg of keratinocyte growth supplement, 0.5 mg/ml of hydrocortisone and 10 % FBS were then added and was incubated at 37 °C and 5 % CO_2_ for 21 days. The media was changed thrice a week until they reached confluence.

### Cytoproliferative effect of denuded human amniotic membrane

To assess the cytoproliferative effects, DHAM were placed on wells of polystyrene tissue culture plates followed by UV sterilization. The wells without HAM were treated as control. RCF, NIH3T3 fibroblasts, A431 keratinocytes, L6 myocytes, and HepG2 hepatocytes were seeded and allowed to grow for different time period as shown in respective figures. For corneal explant culture, the limbal epithelial tissues were cut into multiple pieces and placed on the centre of the wells followed by incubation at 37 °C in a humidified atmosphere containing 5 % CO_2_. In all culture conditions, the medium was renewed every day.

### Distribution morphology of cultivated limbal cells

To observe the distribution morphology of the limbal cells expansion, amniotic membrane grown with cells were harvested after 21 days and subjected to H&E and PAS (Periodic Acid Schiff) staining. The cultures were fixed in 10 percent neutral buffered formalin, processed and embedded in paraffin wax for sectioning and staining. The stained sections were observed under a light microscope.

### Statistical analysis

Results were represented as mean ± s.e.m for at least three experiments. A probability level of *P* < 0.05 was considered statistically significant, and the analysis was done by student’s *t*-test.

## Results and discussion

### General

Decellularised human amniotic membrane (DHAM) is an ECM-like tissue which contains key cell adhesion protein molecules such as fibronectin. Cells including fibroblasts, recognize fibronectin in DHAM via fibronectin-integrin interactions. These stimulated cells in turn secrete fibronectin and assemble an extracellular matrix (Bhatia et al. 2007). The whole process mimics the scaffolding function of the ECM allowing fibroblasts to bind and get stimulated to secrete a variety of growth factors and cytokines that invigorate the wound healing process.

### Denudation of amniotic membrane

In the present work, we have used a combination of non-ionic detergent, alkali and enzymatic treatments for complete decellularisation and preservation of the ECM features in terms of retaining the nativity for optimum cell proliferation, migration and differentiation.

In many decellularisation protocols chelating agents such as ethylenediaminetetraacetic acid (EDTA) aid in cell dissociation from ECM proteins by sequestering metal ions. It is likely that chelating agents contribute to subtle disruptions in protein–protein interactions but they alone are insufficient for superficial cell removal even with agitation, and they are therefore typically used in combination with enzymes such as trypsin or detergents. It is generally preferred to follow a protocol which can retain the structural and functional component of the extracellular matrix. Non-ionic detergents have relatively mild effects upon tissue structure but disrupt lipid–lipid and lipid–protein interactions leaving protein–protein interactions intact. In this regard, we found out that using 0.3 % w/v of non-ionic detergent, which primarily involves the removal of fat and soluble proteins was optimum. Further, to solubilize the cytoplasmic component of the cells including nucleic acids, alkaline/acid treatment was performed and we found sodium peroxide at 0.3 % w/w was optimum as a saponifying and alkaline agent. The surfactant treatment will typically remove about 30 % to about 40 % and the alkaline treatment about 60 % to about 70 % of the non-collagenous protein ultimately. The alkaline treatment opens up the tertiary and secondary structure of collagen, which in turn facilitates easy removal of non-collagenous matter. In the last parameter of enzymatic aided processing, 10 h incubation with trypsin was found to be optimum.

### Estimation of collagen content

Collagen content of the HAM membrane before and after processing was estimated by its hydroxyproline content. The value so obtained was then used to estimate the total collagen per milligram of tissue, assuming that collagen comprises 13.7 % of hydroxyproline by wet weight (Hamlin and Kohn 1971). The content of collagen for unprocessed HAM came out to be 59.8 ± 0.5 %, whereas DHAM showed 83.5 ± 0.4 %. The remaining part of the membrane could be attributed to the presence of other molecules like fibronectin of the ECM. From the results it is evident that the decellularised membrane shows the removal of cellular proteins and other materials while retaining the fibrous collagen giving it more purity compared to the unprocessed one.

### DSC studies

**Fig. 1:**
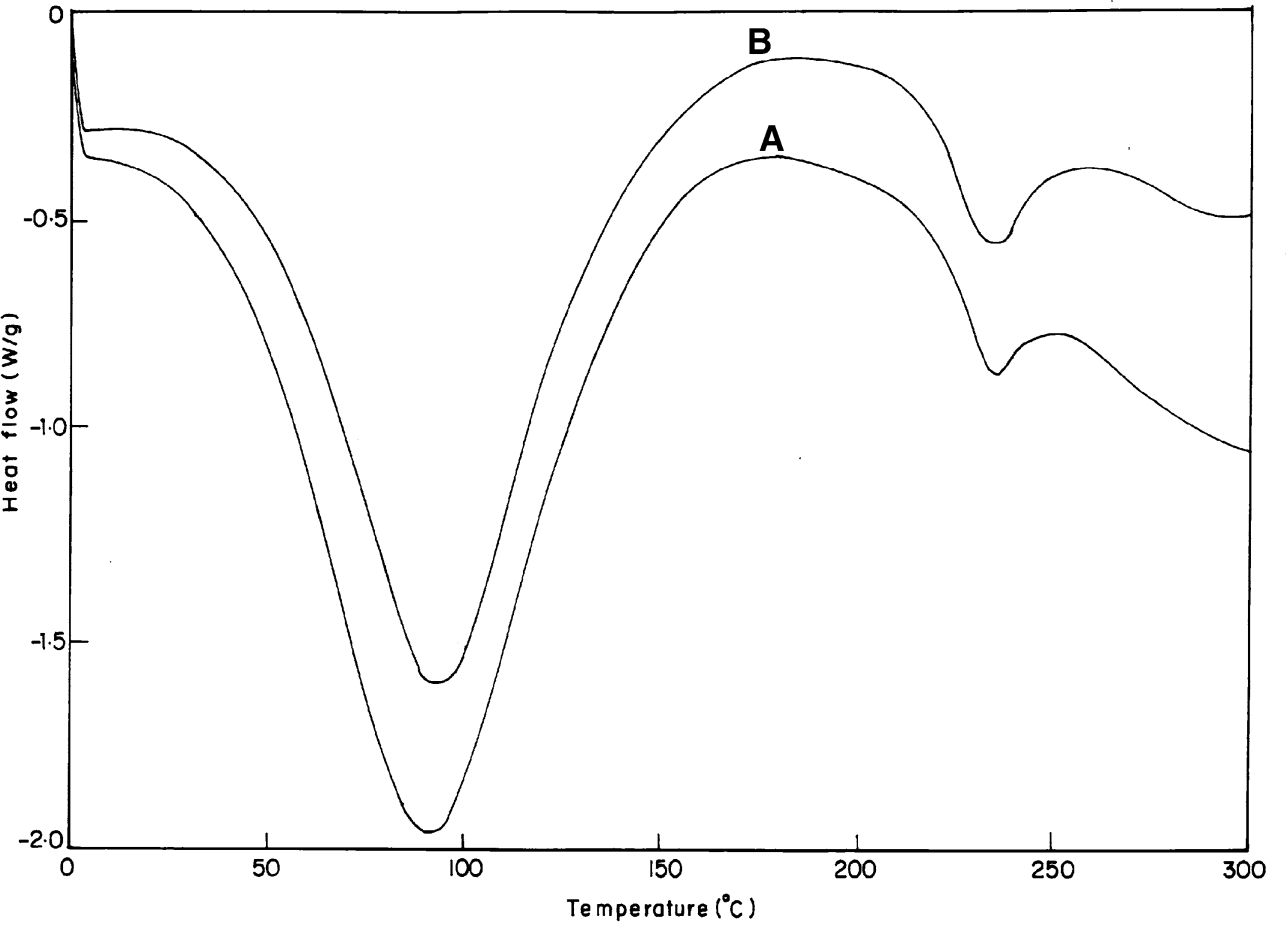
Differential scanning calorimetric scan of unprocessed human amniotic membrane (*curve* *A*) and decellularised human amniotic membrane (*curve* *B*) recorded from 0 to 300 °C using calorimeter

The HAM membranes were analyzed for their thermal stability by DSC and thermograms of the same were shown in (Fig. 1). The first endothermic peaks for both unprocessed HAM (curve A) and DHAM (curve B) were seen to be centred around 94 °C which is associated with the amount of hydrogen-bound water in the matrix (sometimes referred to as the denaturation peak or glass transition; Tg). The unprocessed HAM showed broad Tg peak with more heat absorption, reflecting the complex molecular nature of the matrix compared to DHAM. However, DHAM showed higher Tg value with lesser heat absorption, which resulted from UV cross-linking and collagen dominancy after denudation of the AM membrane. The higher temperature respective exothermic peaks centred around 179 °C corresponding to the crystalline temperatures (Tc) of the membrane. The second respective endothermic peaks at higher temperatures centred around 259 °C, corresponding to the crystalline melting temperature (Tm) of the membranes. In case of DHAM, the Tc and Tm were found to be broader, indicating the differences in distribution of crystal sizes, as post-processing resulted in the removal of many biomolecules from the HAM. However, the kinds and nature of these biomolecules need to be identified and characterized further.

### FTIR studies

**Fig. 2:**
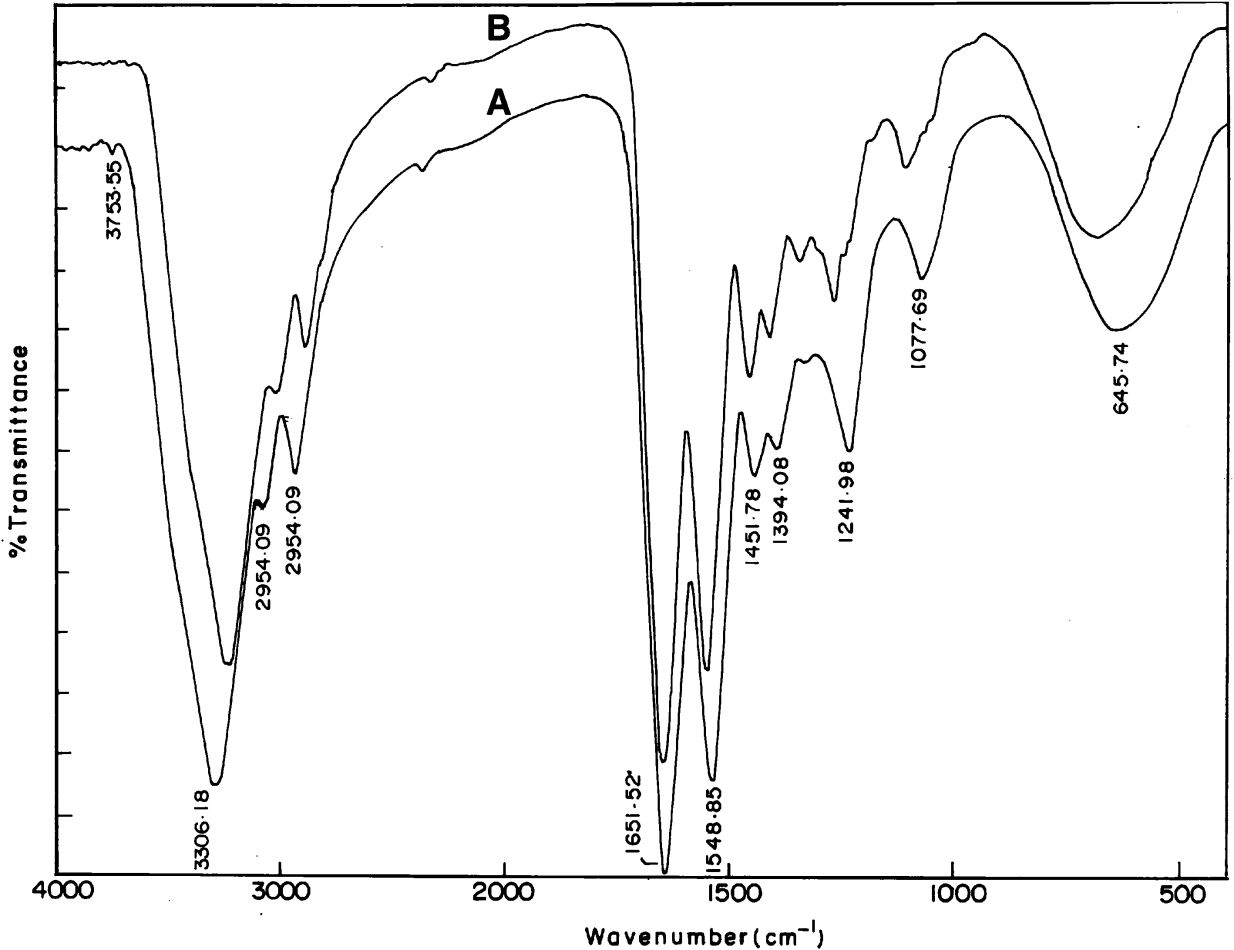
Fourier transform infrared spectrum of unprocessed human amniotic membrane (*curve* *A*) and decellularised human amniotic membrane (*curve* *B*) recorded through spectrometer at 25 °C. The spectra were recorded from 400 to 4000 cm^−1^ and 30 to 100 % transmittance (*T*)

The qualitative natures of the spectra for unprocessed HAM and DHAM are alike with little shift in the peaks due to difference in the matrix nature (Fig. 2). In the spectrum of DHAM (Curve B), seven characteristic absorption bands at the frequencies of 3310, 2959, 1652, 1551, 1453, 1398, 1240, 1079 and 652 cm^−1^ and in case of unprocessed HAM (Curve A) bands at the frequencies of 3306, 2954, 1651, 1548, 1451, 1394, 1241, 1077, and 645 cm^−1^ were observed.

The absorption band around 1600–1640 cm^−1^ corresponds to amide-I protein absorption band and is mainly attributed to C=O stretching mode, and the other absorption band around 1510–1560 cm^−1^ corresponds to amide-II protein absorption band which attributed to N–H bending mode and C–N stretching mode (Socrates 2004). The peaks at around 1210–1300 and 1070–1080 cm^−1^ attributed to protein (amide III) and also to the phosphodiester group of nucleic acids, glyco- and phospho-lipids. The amide III band resulted from in-phase combination of C–N stretching and N–H in-plane bending, with some contribution from C–C stretching and C=O bending vibrations (Grdadolnik 2002). This peak region was comparatively broad in unprocessed HAM (Curve A) which could be due to the presence of cellular components. Peak at 2960 cm^−1^ can be assigned to an asymmetry stretching mode of CH_3_ group. The band close to 1450 cm^−1^ is probably associated with C-H bending modes and the amide-A band (NH stretching) which is observed at 3300–3310 cm^−1^ is almost symmetric, suggesting that the amount of water must be low. The peaks at 1398 and 640–650 cm^−1^ are attributed to the carboxylate ion and the C=O planar deformation vibration of amide IV, respectively (Nivens et al. 1993).

Protein spectra are characterized by amide stretching and bending vibrations. A noticeable shift in the amide II band from 1550 to 1530 cm^−1^ corresponds to the denaturation of collagen helix. This shift highlights an increase in the separation of the amide I and amide II bands at 1650 and 1550 cm^−1^. There is no noticeable shift in the amide peaks in DHAM spectrum, evidencing the nativity of the collagen molecules in the processed membrane.

### Histological analysis

**Fig. 3:**
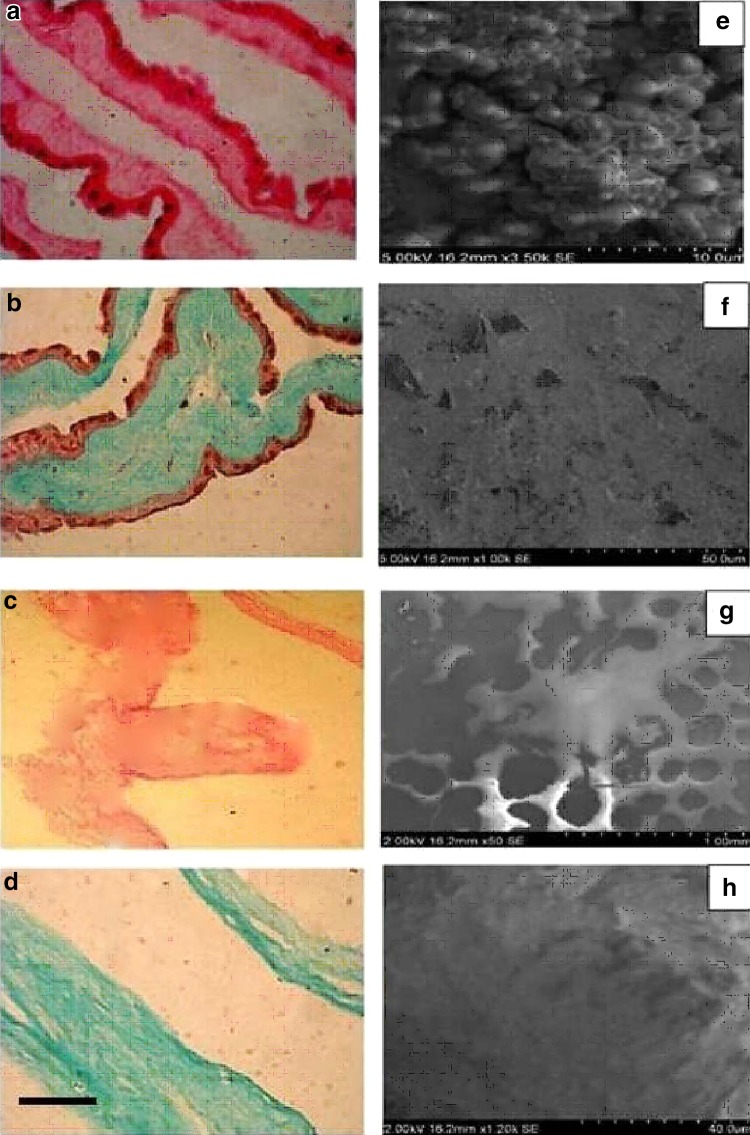
H&E stained sections of unprocessed amniotic membrane (**a**) and decellularised amniotic membrane (**c**). Masson’s Trichrome stained sections of unprocessed amniotic membrane (**b**) and decellularised amniotic membrane (**d**). In **b** and **d** *blue colour* indicates the presence of collagen bundles. (Magnification ×200; *Bar* 50 μm). Scanning electron microscopic picture of human amniotic membrane: **e** unprocessed membrane with amniotic epithelial cells, **f** basement surface of unprocessed membrane, **g** decellularised membrane with the impressions of cellular basement and **h** basement surface of processed membrane

The degree of decellularise process and the integrity of ECM were observed microscopically. Light microscopic studies of the HAM tissue sections stained with hematoxylin and eosin (H&E) indicated that the unprocessed HAM is characterized by an epithelial surface made up of a cobblestone epithelium (Fig. 3a, b) and decellularise/treatment had completely removed the cellular components from the HAM with no apparent disruption of the tissue and fibril structures (Fig. 3c, d). We also observed the five layered structure of unprocessed HAM (Liang et al. 2009) comprising a columnar epithelial cell layer; a basement membrane layer; a contact layer, a mesenchymal cell layer and a spongy layer. Masson’s trichrome staining indicated, no evident disruption in the overall matrix histo-architecture and the major structural component (i.e., collagen) of HAM also appeared to be conserved (Fig. 3d). The portions of the sections which stained blue (dark) confirmed the dominance of collagen. Moreover, the absence of nuclear structures in H&E staining were indicators of effective cell removal during decellularise process.

### Scaning electron microscopy

SEM of HAM shows the presence of amniotic epithelial cells (Fig. 3e) and the smooth basement membrane (Fig. 3f), whereas the DHAM showed the absence of amniotic epithelial cells and gave the evidence for denudation of the membrane with intact extracellular matrix (Fig. 3g) and smooth basement membrane (Fig. 3h).

### Studies of shrinkage temperature

Shrinkage temperature is the temperature at which a biomaterial contracts when immersed in water whose temperature is gradually raised (1 °C/min). The unprocessed HAM showed the shrinkage temperature of 75 ± 0.5 °C whereas DHAM recorded a relatively lower temperature of 73 ± 0.4 °C. This slight difference could be because of collagen content, as its denaturation is a relatively slow process compared to other proteins. This slow denaturation has been attributed to the slower rate of the cis–trans isomerization reaction of prolines, which are present in large amounts in collagen (Privalov 1982). Also, the crosslinking and increased compactness in the processed DHAM might have imparted this feature, as the degree of bound water would also be less.

### Studies of tensile strength

The ability of the AM membrane to bear load was studied by its stress–strain behavior. The mean tensile strength of unprocessed HAM was recorded as 32.27 ± 2.5 kg/cm^2^, whereas the processed DHAM showed 29.7 ± 1.0 kg/cm^2^. The decreased strength of the DHAM may be due to the reduction in the cellular component and the changes in the architecture of the HAM, as the loss of cells would lead to loss of cell adhesion molecules which can also have a role in mechanical strength of the membrane. However, the process of decellularisation did not reduce the strength of the resulting extracellular matrix drastically. The UV crosslinking of the DHAM imparted the higher mechanical strength, as UV unexposed DHAM was found to have lower strength (24.9 ± 1.3 kg/cm^2^).

### Transparency measurements

The transparency measurements of DHAM was performed using UV–Vis spectrophotometer (Table 1). The transparency is the most distinguishing feature needed in corneal shields for ophthalmic applications, which is achieved up to more than 70 % in both (dry and wet) conditions. Moreover, we found more than 98 % transparency at 600 nm in wet condition. This degree of transparency makes this material suitable to be used in the field of ophthalmic applications such as bandage lense, ophthalmic inserts, etc.

**Table 1:** Percentage of Transparency of DHAM calculated using a UV–visible spectrophotometer from 250 to 700 nm in both dry and wet condition with respect to air

nm	Air (% *T*)	(% *T*)	
		DHAM (wet)	DHAM (dry)
300	100.9	78	70
400	100.5	85	75
500	100.5	87	80
600	100.5	98	87
700	100.2	90	88

### Biological characterization

**Fig. 4:**
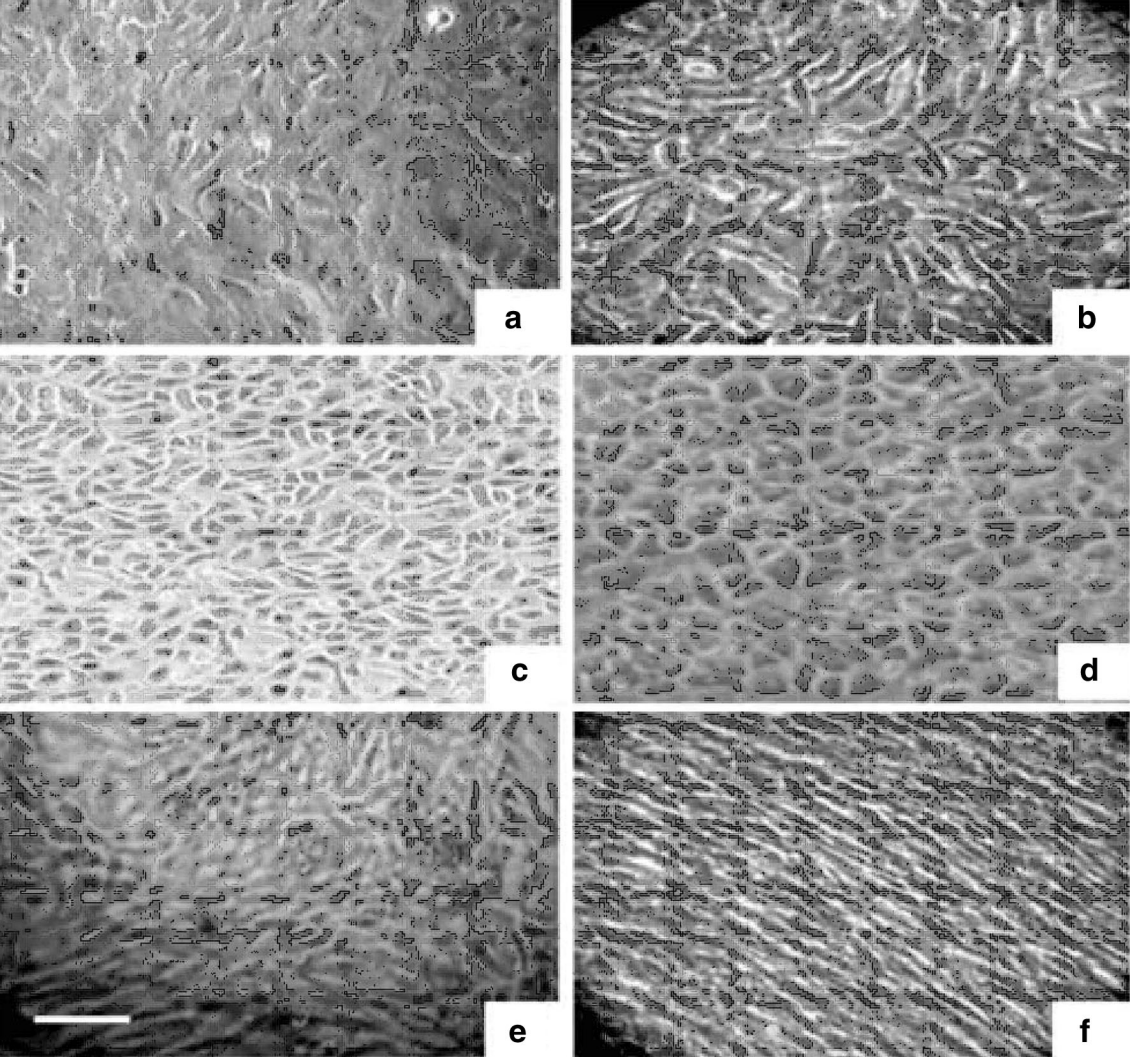
Microscopic images of cellular proliferation on DHAM: **a** rat cardiac fibroblasts, **b** NIH3T3 fibroblasts, **c** A431 keratinocytes, **d** HepG2 hepatocytes **e** L6 myoblasts and **f** well differentiated myoblast into myotubes. (Magnification ×200; *Bar* 40 μm)

The efficacy of the DHAM to support cell proliferation and viability was assessed by growing rat cardiac fibroblasts (Fig. 4a), NIH 3T3 fibroblasts (Fig. 4b), keratinocytes (Fig. 4c), hepatoctes (Fig. 4d) and L6 myoblasts (Fig. 4e, f). Although cells were always incubated for the mentioned time period before the assay were carried out, the number of cells were not exactly reproducible because values caused by each experimental condition varied. Moreover, the doubling time and metabolic status would vary depending on the cell types. The variations resulted in the control must also be due to the difference in the experimental timing, as all the experiments could not be performed simultaneously. Compared to the tissue culture treated polystyrene surface (control), proliferation of all cell types used was significantly upregulated with normal morphologies. Differentiation of myoblasts into myotubes were more pronounced with DHAM (Fig. 4f), as their morphology shows more striped appearance with larger and elongated features. Cessation of proliferation by skeletal muscle precursor cells (myoblasts) coincides with the induction of fusion to form multinucleated structure (myotubes) and the initiation of differentiation, the process through which sarcomeres are formed. The DHAM would have provided the stimulus to myoblasts which in turn modulated the myogenic factors (such as myogenin, meltrins, myocyte enhance factors, serum response factor) which are being required for the fusion and expression of striated alpha-actin genes. The results suggest that DHAM provides a surface that is amenable to cellular adhesion, viability, and functionality. DHAM has been strongly suggested as a potential 3D cell-carrier scaffold for delivery of human adipose-derived stem cells (hADSC), in tissue engineering and regenerative medicine applications (Gholipourmalekabadi et al. 2016). Similar results were reported on culturing fibroblast on decellularised human amniotic membrane (all cells and associated growth factors removed) and showed similar morphologies when the same were cultured on fibronectin (Sakuragawa et al. 2001). A more significant consequence of fibroblast binding to DHAM is the secretion of cytokines, which are involved in recruiting other cells to the site of injury, initiating cell–cell communication and allowing for further progression of the wound healing process. In rat models, MCP-1, a chemotactic cytokine has been examined for its ability to accelerate reepithelization and collagen synthesis in wound healing (Schmidt et al. 1982).

**Fig. 5:**
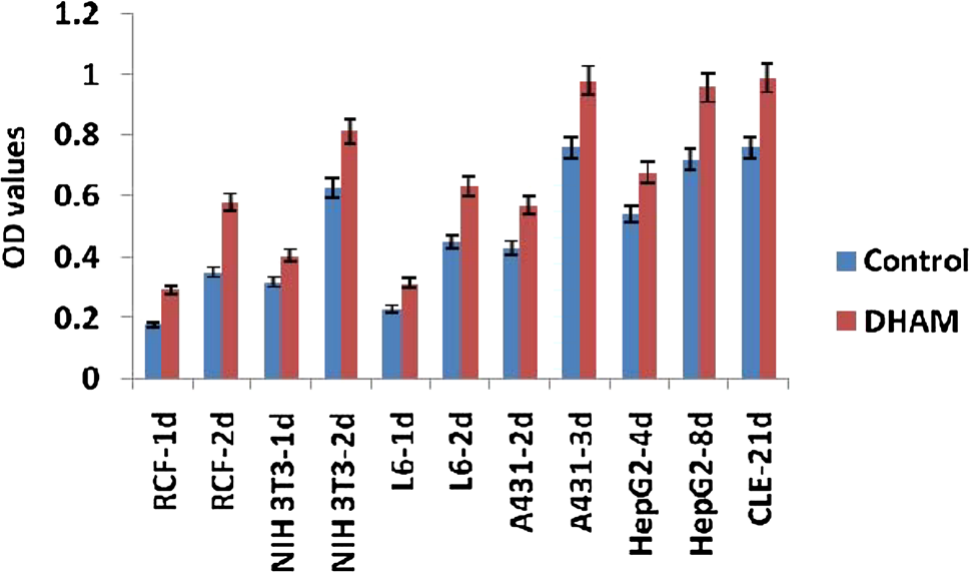
Degree of proliferation measured by MTT assay on NIH 3T3 fibroblasts, rat cardiac fibroblast (RCF), L6 myoblasts, A431 keratinocytes, HepG2 hepatocytes and corneal limbal epithelial (CLE) cell after different incubation period over control and DHAM. The *blue colour bars* represent the control (tissue culture treated polystyrene surface). *Bars* represent mean ± SE of three independent experiments

The overall purpose of this study is to evaluate the feasibility of the modified method to decellularise HAM and to assess the efficacy of the DHAM as a cellular carrier by assessing their proliferation efficiency and maintenance of phenotype in vitro. The proliferating activity of all tested cells in DHAM was found to be up-regulated. The acellular scaffold developed from HAM could be highly suitable for wide applications for tissue engineering in the form of a surgical patch and a cell delivery system. The proliferative efficiency measured by MTT assay was found to be upregulated by 23 % for rat cardiac fibroblasts, 19 % for NIH 3T3 fibroblasts and 21 % for L6 myoblasts after 2 days (Fig. 5). Similarly, the upregulated growths were found to be 25 % for keratinocytes after 3 days and 27 % for hepatocytes (HepG2) after 8 days (Fig. 5). In tissue engineering the effective repair requires remodelling of the extracellular matrix, particularly in the form of new matrix deposition. DHAM is an ECM-like tissue, which is devoid of cells and may contain least or insignificant amount of associated growth factors but contains cell adhesion proteins which can be essential for the binding and proliferation of cells.

### Explant culture of corneal limbal cells

Epithelial migration from limbal biopsies was observed at the end of 48 h in both conditions. The cells further multiplied forming the outgrowth of monolayer and 80–100 % confluent growth was seen on day 21. The cells were harvested and viable count, as estimated by trypan blue exclusion test, ranged from 95 to 98 % with approximate yield of 4 × 10^4^ cells per mm^2^. The expansion of limbal cells over DHAM were more pronounced in comparison to tissue culture treated polystyrene surface, as MTT assay showed 13 % more proliferation on DHAM (Fig. 5).

**Fig. 6:**
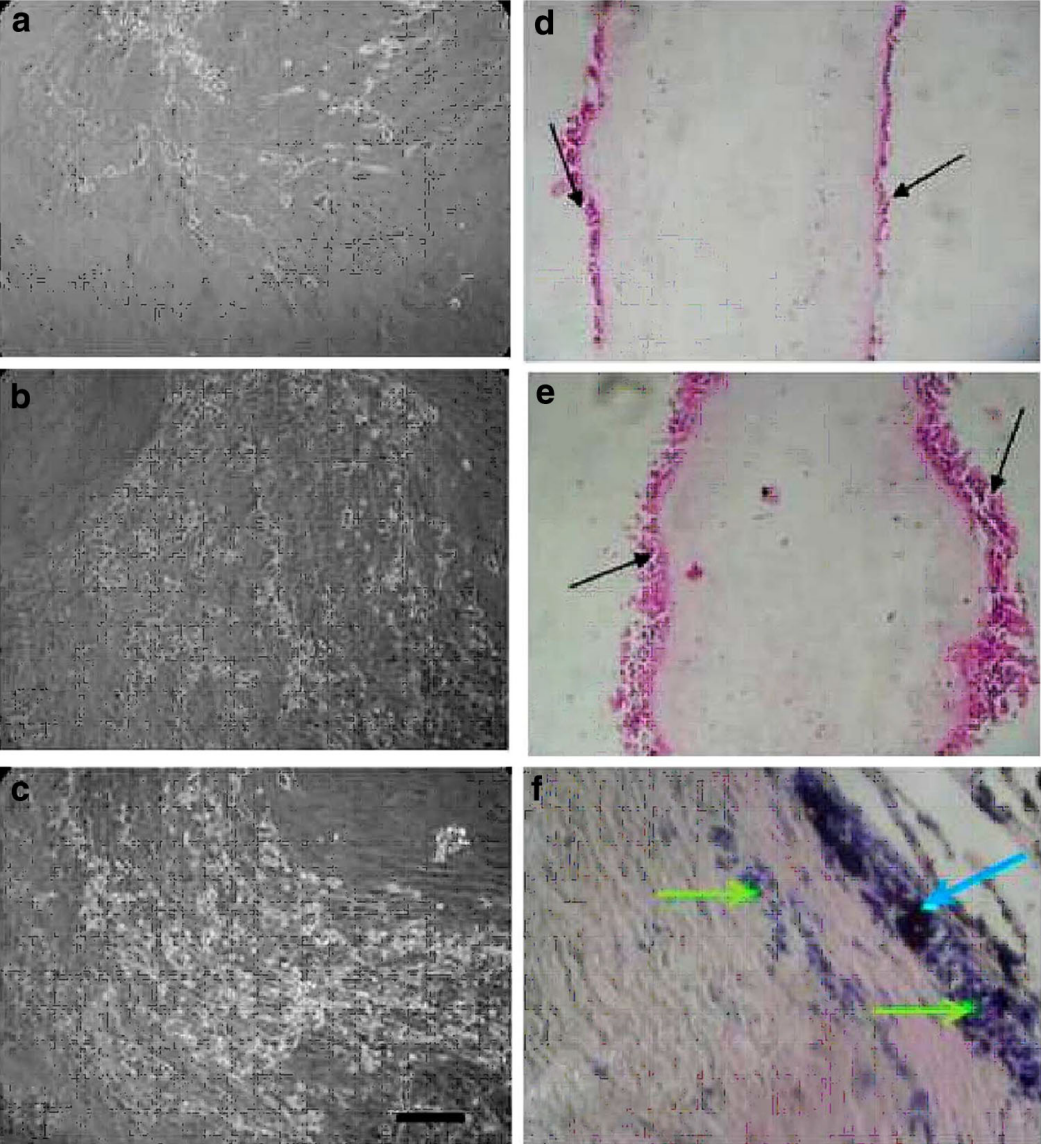
Corneal limbal epithelial cell culture on DHAM: **a** after day 2, **b** after day 6 and **c** after day 21. Histological sections of DHAM after day 21 of limbal cell culture, H&E stained (**d**, **e**) and PAS stained (**f**). (Magnification ×200; *Bar* 50 μm)

The corneal limbal cells grown on DHAM were shown in Fig. 6a–c. However, morphology of the corneal limbal epithelial (CLE) cell was found to be circular at the end of day 2 (Fig. 6a), they further multiplied forming monolayer and found to be 50–60 % confluent as observed on day 6 (Fig. 6b). On day 21, 92–100 % confluent growth was seen on the membrane (Fig. 6c). In the initial stage of growth, the cells exhibited a spindle shape and later changed to a more cuboidal appearance. After day 21 of the culture, the membrane with cells was fixed and subjected to H&E and PAS staining to observe the morphology of the grown limbal cells distribution into the DHAM.

### Morphology of cultivated cells distribution

In H&E staining, the growth of limbal epithelial cells was elevated and the cultured cells showed the formation of multilayer cells rather than a monolayer (Fig. 6d, e). PAS staining techniques are used to demonstrate polysaccharides, neutral mucosubstances and basement membranes primarily in tissue. The fibroblast stained blue (green arrows in Fig. 6f) and epithelial cells stained brown colour (blue arrows in Fig. 6f). Moreover, in both H&E and PAS staining, the fibroblasts were found to be penetrating into the matrix in between the collagen bundles of the DHAM.

So far either preserved HAM has been used as substrate together with limbal epithelial stem cells for treating conditions like limbal stem cell deficient corneas (Kim and Tseng 1995) or HAM alone has been transplanted to act as substrate to the residual limbal stem cell to expand in defective corneal conditions in which limbus is damaged (Tsubota et al. 1999; Anderson et al. 2001). *Ex vivo* expanded limbal epithelial cells on HAM are also capable of restoring the corneal surface with limbal SC deficiency (Tsai et al. 2000; Schwab et al. 2000; Koizumi et al. 2000). In the current study we have provided additional evidence that DHAM could be an ideal matrix for ex vivo preservation and expansion of limbal epithelial SC.

## Conclusion

A decellularised human amniotic membrane, prepared by a novel modified method using a combination of wetting agent, alkali and enzyme, is able to retain its physical and biological properties as a suitable extracellular matrix with good biocompatibility. The decellullarised membrane can be an ideal matrix for ophthalmic and tissue engineering application.
